# Lateral pelvic lymph node dissection after neoadjuvant chemo-radiation for preoperative enlarged lateral nodes in advanced low rectal cancer: study protocol for a randomized controlled trial

**DOI:** 10.1186/s13063-016-1695-4

**Published:** 2016-11-25

**Authors:** Mingtian Wei, Qingbin Wu, Chuanwen Fan, Yan Li, Xiangzheng Chen, Zongguang Zhou, Junhong Han, Ziqiang Wang

**Affiliations:** 1 Department of Gastrointestinal Surgery, State Key Laboratory of Biotherapy and Cancer Center/Collaborative Innovation Center for Biotherapy, West China Hospital, Sichuan University, Chengdu, 610041 People’s Republic of China; 2 Department of Gastrointestinal Surgery, West China Hospital, Sichuan University, Guo Xue Xiang No.37, Chengdu, 610041 People’s Republic of China; 3 State Key Laboratory of Biotherapy and Cancer Center/Collaborative Innovation Center for Biotherapy, West China Hospital, Sichuan University, Chengdu, 610041 People’s Republic of China

**Keywords:** Rectal cancer, Lateral lymph node dissection, Neoadjuvant chemo-radiation, Total mesorectal excision, Recurrence

## Abstract

**Background:**

Lateral lymph node (LLN) metastasis is a major cause of local recurrence of advanced rectal cancer. Although there is much controversy between Western and Eastern countries on whether lateral pelvic lymph node dissection (LLND) or neoadjuvant chemo-radiation (nCRT) is preferable for the treatment of LLN metastases, existing retrospective cohorts mainly focus on all middle/low advanced rectal cancer patients, not the specific individuals with suspicion of LLN metastases. The aim of this trial is to assess the efficacy and safety of LLND for rectal cancer patients with suspicion of LLN metastases.

**Methods:**

This prospective, multicenter, randomized controlled, single-blinded, phase III trial is designed to enroll 512 eligible patients with advanced rectal cancer and preoperative enlarged lateral lymph nodes. The population will be randomly assigned into the solely total mesorectal excision (TME) group or the TME + LLND group after eligible selection. The primary outcomes are to be 3-year local recurrence rate and 3-year disease-free survival, and the secondary outcomes include 3-year overall survival, 1-year sexual and urinary function, and perioperative outcomes.

**Discussion:**

This is the first randomized trial to investigate the efficacy and safety of LLND for advanced low rectal cancer patients with suspicion of LLN metastases; the result is expected to provide new evidence for the treatment of LLN where there is suspicion of metastases in advanced rectal cancer patients.

**Trial registration:**

This trial was registered at ClinicalTrials.gov (identifier NCT02614157) Registered on 24 November 2015.

## Background

Rectal cancer is the third most common cancer and the fourth leading cause of cancer-related deaths worldwide [1]. Local recurrence of middle/low rectal cancer is not only a poor prognostic factor but also a threat of a terrible quality of life. Although universal usage of neoadjuvant chemo-radiation (nCRT) and total mesorectal excision (TME) have decreased local recurrence rates to 5–10%, the ratio of local recurrence has occupied almost 30% of total metastases and recurrence incidences, which heavily limits the therapeutic effect for advanced rectal cancer [2]. Increasing evidence has demonstrated that lateral pelvic lymph node (LLN) metastasis is a major cause of local recurrence of advanced rectal cancer [3]. At the time of diagnosis, 10–25% of patients with advanced rectal cancer have synchronous LLN metastases, which consequently lead to local recurrence and poor overall survival [4, 5].

Regarding the treatment strategy for LLN metastases, there is much controversy between Western and Eastern countries on whether lateral pelvic lymph node dissection (LLND) [6–10] or nCRT [11, 12] is the best treatment option. Eastern countries, especially Japan, favor LLND following TME for the following reasons: (1) the incidence of LLN metastases reaches as high as 10–25%, and 27% of rectal cancer patients who undergo TME solely (without LLND) would develop local recurrence [13–16], (2) the efficacy of LLND equals that of resection of local lymph node metastases. A large cohort of 11,567 cases from Japan demonstrates that resection of metastatic iliac lymph nodes does not show any differences between TME in patients with clinical stage TxN2aM0, and resection of obturator and external iliac lymph nodes favors that of liver metastasis [11], and (3) Japanese guidelines for the treatment of colorectal cancer in 2014 still recommend that patients with stages II/III rectal cancer below the peritoneal reflection undergo regular TME + LLND [17].

On the contrary, Western countries favor nCRT and TME for LLN metastases, holding that: (1) the rate of LLN metastases is relatively low, and LLN metastases are regarded as systemic metastases, (2) LLND involves longer operative time, higher postoperative complications, and results in a poorer quality of life. Therefore, American NCCN and European ESMO guidelines recommend single TME for rectal cancer but, if necessary, LLDN is added when LLN indeed show evidence of metastases.

Despite this, existing retrospective cohorts mainly focus on all middle/low advanced rectal cancer patients, not the specific individuals with suspicion of LLN metastases. And those results indicate almost no differences in local recurrence and overall survival, except for longer operation time, more blood loss, and more perioperative complications for LLND [7–9, 18]. A Korean report enrolled 900 advanced rectal cancer patients with preoperative swollen LLNs, which showed that the recurrence rate was associated with the diameter of the LLNs [16]. However, there is no strict prospective randomized controlled study on the comparison of TME + LLND and TME for low rectal cancer with suspicion of LLN metastases after nCRT. Thus, the objective of this study is to assess the efficacy and safety of LLND for low rectal cancer patients with suspicion of LLN metastases (TME + LLND versus TME). Furthermore, the risk factors (such as radiologic factors, pathologic factors, and serum protein) to predict local recurrence and overall survival will be further investigated.

## Methods

### Study design

**Fig. 1:**
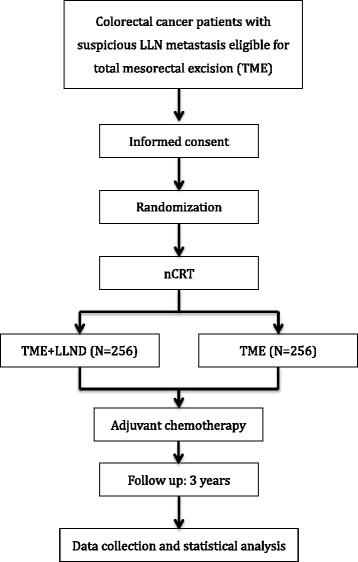
Example template of recommended content for the schedule of enrollment, interventions, and assessments

This is a prospective, multicenter, randomized controlled, single-blinded, phase III trial in which patients will be randomly assigned into two parallel comparison groups (the TME + LLND group and the TME group). The flow diagram of this trial is shown in Fig. 1. Our trial began in May 2016 in the West China Hospital of Sichuan University and is expected to end in 2022.

### Ethics

This trial protocol has been approved by the Ethics Committee of West China Hospital of Sichuan University on 13 May 2016 with protocol number 89, and is registered at ClinicalTrials.gov (identifier NCT02614157). Written informed consent will be obtained from every patient.

### Study population

The inclusion and exclusion criteria of our trial are shown in Table 1. All eligible patients will strictly comply with the criteria.

**Table 1:** The inclusion and exclusion criteria

Inclusion criteria:
•Age (years): 18–75
•Histologically confirmed rectal cancer
•Clinical stage: cTxN1-2M0 or cT3-4N0M0
•Suspicion of LLN metastasis: short-axis diameter of lateral lymph nodes (LLN) >5 mm; short diameter of common iliac or external iliac lymph nodes >6 mm [16, 24, 25]
•No extramesorectal lymph node swelling (short-axis diameter <10 mm)
•No invasion of other organs
•Performance Status (PS): 0, 1
•No past history of chemotherapy, pelvic surgery, or radiation
•Written informed consent operative criteria:
•Mesorectal excision is performed
•Operative findings:
Main lesion of the tumor is located at the rectum
Lower tumor margin is below the peritoneal reflection
Stage R0 after resection
Exclusion criteria:
•High rectal cancer: lower tumor margin is above the peritoneal reflection
•Multiple-cancer patients
•Pregnant patients
•Patients with psychological disorder
•Steroid administration
•Cardiac infarction within previous 6 months
•Severe pulmonary emphysema and/or pulmonary fibrosis
•Physician’s decision to exclude
•Patients with confirmed LLN metastasis: short-axis diameter of LLN >10 mm and a lymph node with an irregular edge, heterogeneous signal, or obvious enlargement and after nCRT has been completed, lymph node enlarges more than 30%
•Emergency surgery
•Patients with coagulopathies

### Randomization and blinding

All eligible participants meeting the inclusion criteria will be randomly allocated into either the TME + LLND group or the TME group. Based on one previous study, randomization is performed using a computer-generated, blocked randomization sequence with a block size of 4 at a 1:1 ratio [19]. The patients will be informed about the treatment details upon admission the day before surgery. Owing to the specificity and ethics, this trial allows the participants, the surgeon, and the investigators to be aware of the whole assignment.

### Intervention

After randomization, all participants will firstly receive a cycle of 5-fluorouracil with oxaliplatin therapy (FOLFOX) or capecitabine (Xeloda) with oxaliplatin therapy (XELOX). Subsequently, radiotherapy with 50.4 Gy is administered in 28 separate doses. Next, three cycles of FOLFOX or XELOX will be continued. About 3 to 4 weeks later, patients allocated to the TME group will undergo standard TME surgery solely, while patients allocated to the TME + LLND group will undergo standard TME surgery plus LLND. The extent of LLND includes bilateral LLND (lymph node groups 263P, 263D, and 283). If the short-axis diameter of the external iliac or common iliac lymph nodes is beyond 6 mm, lymph node groups 293, 273, and 280 will be dissected. Unilateral LLND will be done only when the center of the rectal tumor is limited to a single side (not beyond the midline) and the contralateral lymph nodes are not enlarged. Adjuvant chemotherapy, consistent with the preoperative scheme in every patient, will be continued until reaching eight cycles.

### Outcomes

The primary outcomes of this trial are the 3-year local recurrence rate and 3-year disease-free survival (DFS). The local recurrence is defined as recurrent disease in the pelvis or at the incision and DFS is defined as the time of surgery to the recurrence or end of follow-up. It is diagnosed using the following methods: (1) histological confirmation by biopsy, (2) confirmation by positron emission tomography-computed tomography (PET-CT), (3) CT/magnetic resonance imaging (MRI) shows that there is a mass in the pelvis which is growing with/without symptoms.

The secondary outcomes include 3-year overall survival (OS) and disease-free survival (DFS) at 3 years post surgery, sexual and urinary function at 1 year post surgery, and perioperative outcomes. OS is defined as the time of surgery to the date of death from any cause or the date of follow-up. Sexual function for men and women is assessed by using the International Index of Erectile Function-5 scoring system (IIEF-5) and the Female Sexual Function Index (FSFI), respectively [20, 21]. Urinary function is assessed by using the International Prostate Symptom Score (IPSS) [22]. Perioperative outcomes include operation time, intraoperative blood loss, intraoperative transfusion, incision length, intraoperative complications, time to first flatus, time to liquid diet intake, postoperative hospitalization days, postoperative complications, and mortality.

### Follow-up

All participants will be followed up regularly as per schedule (Table 2). Biopsy or PET-CT would be done when it is necessary to diagnose recurrence.

**Table 2:** Trial schedule

Measures	M1	M3	M6	M9	M12	M18	M24	M30	M36
Physical examination	×	×	×	×	×	×	×	×	×
Blood test	×	×	×	×	×	×	×	×	×
CEA	×	×	×	×	×	×	×	×	×
CA 19-9	×	×	×	×	×	×	×	×	×
CT of chest			×		×	×	×	×	×
CT/MRI of abdomen and pelvis			×		×	×	×	×	×
Colonoscopy			×		×	×	×	×	×
IIEF-5	×	×	×	×	×				
FSFI	×	×	×	×	×				
IPSS	×	×	×	×	×				

*CA* cancer antigen, *CEA* carcinoembryonic antigen, *CT* computed tomography, *FSFI* Female Sexual Function Index, *IIEF* International Index of Erectile Function, *IPSS* International Prostate Symptom Score, *M* month*, MRI* magnetic resonance imaging

### Data collection and management

We have invited more than 10 large medical centers, which have developed experienced LLND surgery in China, to participate in this trial. We have made universal standards including inclusion and exclusion criteria, nCRT, and TME + LLND standard for all units. In order to obtain good-quality monitoring, we have hired a professional clinical research company to organize and manage the work of all participant units. A Case Report Form is made for each participant to record their data.

### Dropping out

When this trial begins, a participant can drop out of this trial even if written informed consent has been signed if: (1) the participant decides to refuse for any reason, (2) unacceptable adverse events are experienced, (3) an investigator withdraws the participant for that participant’s benefit, (4) a participant receives other treatment, such as biological immune therapy, which is not approved in this trial, (5) a participant becomes pregnant in the process of this trial, and (6) the compliance of the participant is poor.

### Safety and adverse effect

All adverse events must be carefully recorded and reported, including nCRT complications and perioperative complications. All severe adverse events in this trial should be reported every 6 months and adverse events which are life-threatening should be recorded and reported within 24 h.

### Sample size and statistical analysis

Our trial aims to demonstrate that LLND could decrease the 3-year local recurrence rate of low rectal cancer patients with suspicion of LLN metastases. Based on previous studies, we assume that the 3-year local recurrence rate for patients with suspicion of LLN metastases who do not undergo LLND is 15% [13–16], and LLND is expected to show a reduction of 7 percentage points. G*Power 3.1 is used to calculate the sample size. A statistical power of 0.80 and a significance level of 0.05 are chosen, and we assume that there will be a 10% dropout rate. Therefore, 512 patients are needed in this specific trial (256 patients in each group).

All participants in this trial will be analyzed using the intention-to-treat analysis. The chi-square test and Student’ *t* test will be used to compare categorical data and continuous data for the two groups, respectively. The 3-year local recurrence rate, the primary endpoint of this trial, will be analyzed using the Kaplan-Meier method, as well as OS and DFS. In addition, based on different characteristics (such as age, sex, tumor stages, etc.), which can identify the influence of competing risk factors, the Cox proportional hazards (PH) regression model will be tested if Schoenfeld Residuals Analysis confirms PH assumption validation. Otherwise, an alternative extended Cox PH model will be used. SPSS 21.0 (SPSS Inc., Chicago, IL, USA) will be used for all statistical analyses, and a *P* value < 0.05 is considered significant.

## Discussion

Currently, the hot issue of the treatment of LLN in advanced rectal cancer patients is still being debated in different regions. Eastern countries, especially Japan, favor LLND following TME; however, Western countries believe that nCRT and TME is recommended, and LLDN is added only when a LLN is preoperatively diagnosed as metastatic. Actually, preoperatively enlarged LLNs are not common and only a small proportion of LLDs with a maximum short-axis diameter ≥10 mm, or with a spiculated or indistinct border, or a mottled heterogenic pattern are diagnosed as metastatic [23]. The remaining large proportion of LLNs are underdiagnosed as being metastatic and often confuse surgeons’ treatment strategy. Thus, our trial pays particular attention to this stratification of suspicious LLNs, assessing the efficacy and safety of the LLND in advanced rectal cancer patients.

Another issue we have considered in our trial is the randomization time. In our trial, we randomly allocated participants before nCRT rather than after nCRT. Because LLN metastases are commonly diagnosed before nCRT by their features on pelvic MRI or CT and when chemo-radiation has achieved its effect, the treatment-sensitive regions of any enlarged LLNs will have decreased in size which may confuse the randomization if allocation is performed after nCRT.

This trial is the first randomized controlled trial to investigate the role of LLND in reducing local recurrence rates for low advanced rectal cancer patients with suspicion of LLN metastases. If the efficacy of LLND is verified, which reduces the local recurrence rate from 15% to 8%, LLND can be recommended as a new standard strategy for low advanced rectal cancer patients with suspicion of LLN metastases.

### Trial status

This trial was initiated in May 2016 and is currently enrolling patients.

## References

[1] Ferlay J, Soerjomataram I, Dikshit R, Eser S, Mathers C, Rebelo M (2015). Cancer incidence and mortality worldwide: sources, methods and major patterns in GLOBOCAN 2012. Int J Cancer..

[2] Baik SH, Kim NK, Lee YC, Kim H, Lee KY, Sohn SK (2007). Prognostic significance of circumferential resection margin following total mesorectal excision and adjuvant chemoradiotherapy in patients with rectal cancer. Ann Surg Oncol..

[3] Quadros CA, Falcao MF, Carvalho ME, Ladeia PA, Lopes A (2012). Metastases to retroperitoneal or lateral pelvic lymph nodes indicated unfavorable survival and high pelvic recurrence rates in a cohort of 102 patients with low rectal adenocarcinoma. J Surg Oncol..

[4] Ueno M, Oya M, Azekura K, Yamaguchi T, Muto T (2005). Incidence and prognostic significance of lateral lymph node metastasis in patients with advanced low rectal cancer. Br J Surg..

[5] Yano H, Moran BJ (2008). The incidence of lateral pelvic side-wall nodal involvement in low rectal cancer may be similar in Japan and the West. Br J Surg..

[6] Ueno H, Mochizuki H, Hashiguchi Y, Ishiguro M, Miyoshi M, Kajiwara Y (2007). Potential prognostic benefit of lateral pelvic node dissection for rectal cancer located below the peritoneal reflection. Ann Surg..

[7] Sugihara K, Kobayashi H, Kato T, Mori T, Mochizuki H, Kameoka S (2006). Indication and benefit of pelvic sidewall dissection for rectal cancer. Dis Colon Rectum..

[8] Yano H, Saito Y, Takeshita E, Miyake O, Ishizuka N (2007). Prediction of lateral pelvic node involvement in low rectal cancer by conventional computed tomography. Br J Surg..

[9] Georgiou P, Tan E, Gouvas N, Antoniou A, Brown G, Nicholls RJ (2009). Extended lymphadenectomy versus conventional surgery for rectal cancer: a meta-analysis. Lancet Oncol..

[10] Nagawa H, Muto T, Sunouchi K, Higuchi Y, Tsurita G, Watanabe T (2001). Randomized, controlled trial of lateral node dissection vs. nerve-preserving resection in patients with rectal cancer after preoperative radiotherapy. Dis Colon Rectum.

[11] Kim JC, Takahashi K, Yu CS, Kim HC, Kim TW, Ryu MH (2007). Comparative outcome between chemoradiotherapy and lateral pelvic lymph node dissection following total mesorectal excision in rectal cancer. Ann Surg..

[12] Watanabe T, Tsurita G, Muto T, Sawada T, Sunouchi K, Higuchi Y (2002). Extended lymphadenectomy and preoperative radiotherapy for lower rectal cancers. Surgery..

[13] Suzuki K, Muto T, Sawada T (1995). Prevention of local recurrence by extended lymphadenectomy for rectal cancer. Surg Today..

[14] Sato H, Maeda K, Maruta M, Masumori K, Koide Y (2006). Who can get the beneficial effect from lateral lymph node dissection for Dukes’ C rectal carcinoma below the peritoneal reflection?. Dis Colon Rectum..

[15] Kim TH, Jeong SY, Choi DH, Kim DY, Jung KH, Moon SH (2008). Lateral lymph node metastasis is a major cause of locoregional recurrence in rectal cancer treated with preoperative chemoradiotherapy and curative resection. Ann Surg Oncol..

[16] Kim MJ, Kim TH, Kim DY, Kim SY, Baek JY, Chang HJ (2015). Can chemoradiation allow for omission of lateral pelvic node dissection for locally advanced rectal cancer?. J Surg Oncol.

[17] Watanabe T, Itabashi M, Shimada Y, Tanaka S, Ito Y, Ajioka Y (2015). Japanese Society for Cancer of the Colon and Rectum (JSCCR) guidelines 2014 for treatment of colorectal cancer. Int J Clin Oncol..

[18] Kusters M, Marijnen CA, van de Velde CJ, Rutten HJ, Lahaye MJ, Kim JH (2010). Patterns of local recurrence in rectal cancer; a study of the Dutch TME trial. Eur J Surg Oncol..

[19] Hu X, Fang Y, Li H, Liu W, Lin S, Fu M (2014). Protocol for seizure prophylaxis following intracerebral hemorrhage study (SPICH): a randomized, double-blind, placebo-controlled trial of short-term sodium valproate prophylaxis in patients with acute spontaneous supratentorial intracerebral hemorrhage. Int J Stroke..

[20] Rosen RC, Cappelleri JC, Smith MD, Lipsky J, Pena BM (1999). Development and evaluation of an abridged, 5-item version of the International Index of Erectile Function (IIEF-5) as a diagnostic tool for erectile dysfunction. Int J Impot Res..

[21] Rosen R, Brown C, Heiman J, Leiblum S, Meston C, Shabsigh R (2000). The Female Sexual Function Index (FSFI): a multidimensional self-report instrument for the assessment of female sexual function. J Sex Marital Ther..

[22] Barry MJ, Fowler FJ, O'Leary MP, Bruskewitz RC, Holtgrewe HL, Mebust WK (1992). The American Urological Association Symptom Index for benign prostatic hyperplasia. The Measurement Committee of the American Urological Association. J Urol.

[23] Kim JH, Beets GL, Kim MJ, Kessels AG, Beets-Tan RG (2004). High-resolution MR imaging for nodal staging in rectal cancer: are there any criteria in addition to the size?. Eur J Radiol..

[24] Lim SB, Yu CS, Kim CW, Yoon YS, Park SH, Kim TW (2013). Clinical implication of additional selective lateral lymph node excision in patients with locally advanced rectal cancer who underwent preoperative chemoradiotherapy. Int J Colorectal Dis..

[25] Grubnic S, Vinnicombe SJ, Norman AR, Husband JE (2002). MR evaluation of normal retroperitoneal and pelvic lymph nodes. Clin Radiol..

